# Progress in Surgery

**Published:** 1896-11-14

**Authors:** 


### Progress in Surgery.

### SURGERY OF THE SKULL.

**Hyperostosis Cranii.**—In a very full and instructive paper, Putnam (*Amer. Jour. Med. Sc.*, July, 1896) describes, with illustrations, five new cases of well marked hyperostosis cranii which have come under his own observation. This disease has been described under various names by different observers, *e.g.*, megalo-cephalie (Starr), leontiasis ossea (Virchow), diffuse hyperostosis (Kalindero). It is apparent, from reading the descriptions of the various published cases, that different conditions are at present described under this term. In some cases the disease is diffuse, affecting more or less all the bones of the skull, including the lower jaw; in others it is in the form of osteophytic outgrowths, and is confined to small definite areas, sometimes the lower jaw alone being involved. In some cases it is suppurative, in others sclerotic in character. Its association with rheumatism or syphilis is at present difficult to determine; and whether it is essentially inflammatory or neoplastic in nature is also uncertain. Heredity seems to be strongly indicated as a factor in some of the cases.

The number of complete clinical histories of cases of the disease is as yet small, many of the specimens described having been found in museums, dissecting rooms, &c. From a study of the available histories Putnam gives the chief clinical features of the condition. In the majority of cases the upper part of the face is most involved in the change, while in others it is the vault or the lower jaw which is abnormal. The age at which the disease first makes its appearance is indicated in the following table :—

| | | | |
|---|---|---|---|
| Under 5 years | 2 cases | 20 to 30 years ... | 1 case |
| 5 to 10 „ ... | 1 case | 30 to 40 „ ... | 3 cases |
| 10 to 20 „ ... | 4 cases | Over 40 „ ... | 3 cases |

Before any evident change in the bones has been observed certain signs and symptoms of disease have been noted, such as epileptic attacks, suppuration of the ears, suppuration of tear ducts, erysipelas of the head, and in some cases the bone changes have followed on injuries to the head, *e.g.*, in a case reported by Hale White (*B. M. J.*, June 6th, 1896), as well as in others quoted by Putnam. It is probable that some of these conditions were the result, rather than the cause of the disease of the bones. Headache, due to bone mischief rather than to increased intra-cranial tension, is present in about half of the cases. It is sometimes diffuse, sometimes localised, and it may be associated with pain in the face and limbs. Optic neuritis, or atrophy, has been observed in some cases, but not in all. Nystagnus was present in Hale White's case. Exophthalmos is frequent.

Mental irritability or enfeeblement is comparatively common, and loss of hearing, smell, or taste, when tested for, are usually detected. In Ede's (*Amer. Jour. Med. Sc.*, July, 1896) case hearing was perfectly good. Facial paralysis is sometimes present, and is due to encroachment on the Aqueductus Fallopii. Insecurity in walking and awkwardness in the use of the hands have been noticed in several cases. Drowsiness is sometimes very marked. In one case the teeth fell out quite early, thus opposing Baumgarten's statement that the loss of the teeth is a diagnostic point in favour of sarcoma as against hyperostosis cranii.

The prognosis is always unfavourable, the disease progressing slowly and intermittently, and death resulting from intra-cranial pressure, paralysis, or exhaustion from suppuration.

In many points the clinical history resembles that of cerebral tumour, and the question of operation arises, but from the diffuse nature of the disease and its progressive character, this is not a method of treatment which is often to be advised. Horsley (*Practitioner*, Vol. II., New Series) has met with four cases in which the disease was sufficiently localised to warrant operation. Thyroid treatment has been tried without benefit.

**Tuberculosis perforans.**—A case of this disease is reported by Wallace (*Edin. Med. Jour.*, July, 1896) in a man twenty-three years of age, who, after having suffered from tubercular disease of the ankle and strumous dactylitis of the little finger, developed a painless fluctuating tumour on the right side of the head, just over the anterior part of the temple. On incising the abscess a sequestrum the size of a florin, consisting of the whole thickness of the skull, was found quite loose. A collection of pus separated the dura from the skull for an area of about two inches all round. The edges of the opening in the skull were perfectly smooth. There were no cerebral symptoms at all, doubtless because of the situation (over the frontal lobes), the slow formation of the abscess, and the opening in the cranium. The wall of the cavity was scraped, iodoform gauze was used as a stuffing, and the patient soon recovered.

**Craniectomy.**—Since Lannelongue, in 1890, first suggested the possibility of bringing about an improvement in the mental condition of idiots by the removal of segments of the skull, and so permitting a freer development of the brain, numerous observations have been recorded, but the results have, on the whole, been very disappointing. Telford-Smith ((*Amer. Jour. Med. Sc.*, June 1896) reports the after history of two cases. The first was a fair test case, microcephaly being the only apparent cause of the mental deficiency. An ample amount of skull was removed, the patient was subsequently specially trained and educated, but the result was most meagre, and no more than could be accounted for by the education of the boy. The other case was not one of microcephaly, but one of congenital idiocy. The operation was followed by no improvement whatever. Robertson (*Amer. Med. Surg. Bulletin*, Aug., 1896) also condemns these operations as unjustifiable. Dana and Putnam (*N.Y. Med. Jour.*, Mar., 1896), however, take a more hopeful view of the operation, both having seen "improvement" follow it.

**The Treatment of Bone Defects of the Skull.**—Bone defects due to trauma, suppurative neurosis, or tumour, may be repaired by one of two methods, viz., auto-plastic or hetero-plastic. Von Eiselsberg (*Verhandlungen der Deut. Gessel f. Chirurg.*, 1895) has operated on eight cases. Five of the cases were following operations for traumatic epilepsy. Four were treated by auto-plastic operation and one by means of a celluloid plate, and in all successfully. Tuberculosis was the cause of the deficiency in three of the cases. Two were treated with celluloid plates, but in one the plate had to be removed; in the other, as also in a case treated by auto-plastic operation, the results were satisfactory. Eiselsberg considers that the danger of suppuration, both immediate and remote, is an important objection to the hetero-plastic method. In favour of it are the facts that it is easily carried out, especially in young subjects, is almost bloodless, and that no adhesions form between the dura and the plate introduced, a point insisted upon by Fränkel (*Verhandlungen der Deut. Gessel f. Chirurg.*, 1895.) As regards the technique of the operation, he states: (1) That celluloid is the best material to use; (2) that the plate should be fixed into the diploe; (3) that the plate should always be introduced at a secondary operation; (4) the skin should be lax and sutured without drainage.

Meyer (*N.Y. Med. Jour.*, Mar., 1896) has a favourable opinion of this method of closing apertures in the skull; but Angel (*N.Y. Med. Jour.*, Mar., 1896) is of opinion that the dense fibrous tissue which fills in the gap is sufficient protection.

**Exploration of the Brain with a Needle and Syringe through Capillary Holes Drilled through the Skull.**—Sonchon (*N.Y. Med. Record*, May, 1896) advocates the exploration of the brain in cases of doubtful diagnosis by means of a small exploring needle passed through holes bored in the skull. He claims that it is simpler and as certain as trephining. Hæmorrhage has not been found to be a risk. In a discussion on this paper Manley, Senn, and Fergusson expressed doubts as to the value of the procedure.

